# Integrating Yield Stability and Gross Revenue: Multi-Year Evaluation of *Coffea canephora* Genotypes

**DOI:** 10.3390/plants15132083

**Published:** 2026-07-03

**Authors:** Alana Mara Kolln, Rafael Nunes de Almeida, Rodrigo Barros Rocha, Fábio Luiz Partelli, Alexsandro Lara Teixeira, Larissa Fatarelli Bento de Araújo, Marcelo Curitiba Espindula

**Affiliations:** 1 Federal Institute of Education, Science and Technology of Rondônia (IFRO), Campus São Miguel do Guaporé, São Miguel do Guaporé 76932-000, RO, Brazil; alana.kolln@ifro.edu.br; 2 Legal Amazon Biodiversity and Biotechnology Network (Bionorte), Federal University of Rondônia (UNIR), Porto Velho 76801-059, RO, Brazil; 3 Institute of Research, Technical Assistance and Rural Extension of Espírito Santo (INCAPER), Vitória 29052-010, ES, Brazil; rafael.almeida@incaper.es.gov.br (R.N.d.A.); rodrigo.rocha@embrapa.br (R.B.R.); alexsandro.teixeira@embrapa.br (A.L.T.); 4 Embrapa Café, Brazilian Agricultural Research Corporation (EMBRAPA), Brasília 70770-901, DF, Brazil; 5 Department of Agricultural and Biological Sciences (DCAB), Northern Espírito Santo University Center (Ceunes), Federal University of Espírito Santo (UFES), São Mateus 29932-900, ES, Brazil; partelli@yahoo.com.br; 6 Embrapa Rondônia, Brazilian Agricultural Research Corporation (EMBRAPA), Porto Velho 76815-800, RO, Brazil; larissafatarelli@gmail.com; 7 Rondônia Foundation for Support to the Development of Scientific and Technological Actions and Research (FAPERO), Porto Velho 76801-156, RO, Brazil

**Keywords:** biennial bearing, clonal selection, Conilon coffee, economic return, genotype × environment interaction, gross revenue, robusta coffee, yield stability

## Abstract

Agricultural research helps us to reduce the risks associated with climate variability, pests and diseases, market fluctuations, and biennial bearing. However, genotype characterization is often conducted independently of economic performance and yield stability, limiting the identification of genotypes that combine high yield, stability, and economic return potential. This study characterized the genotype × harvest season interaction and gross revenue of *Coffea canephora* clones evaluated in Rondônia, Brazil, over five harvest seasons (2021–2025). Twenty-eight genotypes were evaluated in a randomized complete block design with four replications and five plants per plot, and yield stability was assessed using the centroid method. Cumulative yield over five harvest seasons totaled 306.22 bags ha^−1^, with a mean of 61.24 bags ha^−1^ per harvest season. The genotype × harvest season interaction revealed distinct temporal yield patterns. Genotypes closest to the ideotype of maximum yield and stability included BAG19, BRS1216, GJ8, GJ25, AS2, and BAG24. Gross revenue simulations based on contrasting historical coffee price scenarios indicated the economic superiority of these clones. Differences in yield potential contributed more to gross revenue variation than biennial bearing. Relative to the overall mean of the 28 genotypes, these clones achieved an 85% increase in mean yield and a 61.2% increase in mean gross revenue.

## 1. Introduction

Coffee production is strongly influenced by external factors such as climate variability, pest and disease incidence, and fluctuations in commodity prices [1,2]. In this context of uncertainty, agricultural research plays a fundamental role in reducing risks and producer vulnerability, thereby contributing to the sustainability and resilience of coffee production systems.

Temporal fluctuations in coffee yield are commonly associated with biennial bearing, a physiological phenomenon characterized by alternating years of higher and lower production, caused by the balance between intense fruiting in one year and reduced branch and flower emission in the subsequent cycle [3]. Due to this competition for assimilation between fruit production, vegetative growth, and the formation of flower buds, clones may differ in their yield distribution across harvest seasons.

Although less pronounced than in *Coffea arabica*, yield oscillation in *Coffea canephora* remains an important agronomic issue and is often analyzed independently of market conditions. For growers, differences in productive stability among clones may affect the regularity of production and, consequently, the economic return obtained over time. Under these conditions, the economic performance of a clone depends not only on cumulative yield, but also on how production is distributed across harvest seasons.

The study of biennial bearing can be approached from a plant genetics perspective through the interpretation of estimates of repeatability, adaptability, and stability of clones [4]. Repeatability corresponds to the proportion of phenotypic variation attributed to genotypic variance and permanent environmental effects. Adaptability refers to the ability of clones to express higher yield in favorable years, whereas stability is associated with the capacity to maintain predictable performance in years of lower productivity, thereby reducing undesirable oscillations [5]. The joint analysis of these parameters can be performed using multivariate approaches that integrate performance over time with previously defined behavioral ideotypes [5,6].

Recent fluctuations in Robusta coffee prices, driven by changes in supply, climate anomalies, and international market dynamics, have increased the uncertainty associated with coffee production. Because prices vary over time, genotypes may differ in gross revenue depending on how production is distributed across harvest seasons. Consequently, yield stability influences not only production consistency but also economic return. Under these conditions, evaluating genotype performance under contrasting price scenarios may help identify materials capable of providing greater economic security and more stable returns over time.

In addition to genetic performance, fluctuations in yield can also be evaluated in terms of gross revenue, as this represents one of the main criteria guiding producers’ decisions [7,8]. In this context, the consideration of different price scenarios—such as stable prices, upward trends, gradual declines, or the occurrence of price spikes—allows the economic performance of genotypes to be assessed under contrasting market conditions. This approach connects two dimensions that are often analyzed separately, enabling the identification of genotypes that remain competitive under different market conditions and supporting more secure coffee production planning.

In this study, clones of *C. canephora* were evaluated over five consecutive harvest seasons, considering genetic performance and gross revenue under different price scenarios. The integration of these approaches seeks to jointly assess the productive consistency of genotypes over time and their potential economic return under contrasting market conditions.

Thus, the objective of this study was to characterize the genotype × harvest season interaction and the associated gross revenue of *C. canephora* clones over five harvest seasons, aiming to identify genotypes that combine high yield, productive stability, and economic security under contrasting coffee price scenarios.

## 2. Results

**Figure 1:**
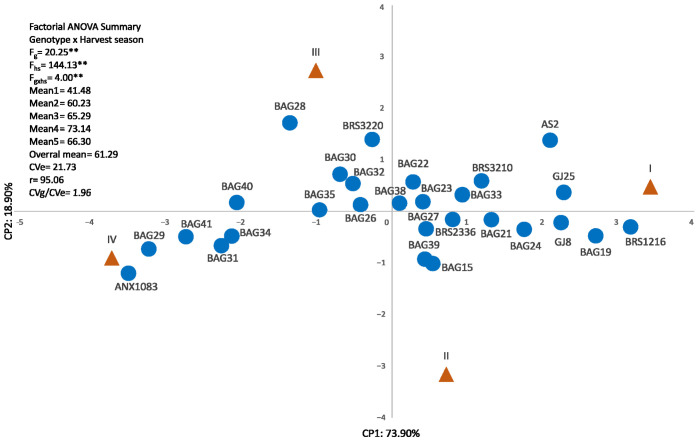
Principal component analysis (PCA) of yield performance of Coffea canephora genotypes evaluated over five harvest seasons. Circles represent the evaluated genotypes, whereas triangles represent ideotypes with contrasting yield patterns: I—maximum yield in all harvests; II—maximum yield in the first, third, and fifth harvests and minimum yield in the second and fourth; III—minimum yield in the first, third, and fifth harvests and maximum yield in the second and fourth; IV—minimum yield in all harvests. In the figure, CVg = genotypic coefficient of variation, CVe = experimental coefficient of variation, CVg/CVe = ratio between genotypic and experimental coefficients of variation, H^2^ = broad-sense heritability, and r = repeatability coefficient. ** indicates significance at the 1% probability level by the F-test.

The evaluation of yield over five harvest seasons resulted in a cumulative production of 306.22 bags ha^−1^, with a mean of 61.24 bags ha^−1^ per harvest season. The relative contribution of each harvest ranged from 14% to 24%, with the second harvest being the most productive, at 73.13 bags ha^−1^ (24%), followed by the fifth harvest (66.10 bags ha^−1^; 22%) and the third harvest (65.29 bags ha^−1^; 21%). The first harvest contributed 60.23 bags ha^−1^ (20%), whereas the fourth showed the lowest yield, with 41.47 bags ha^−1^ (14%) (Figure 1). These results demonstrate the occurrence of yield variability across harvest seasons, reflecting biennial bearing and environmental effects.

The genotype × harvest season interaction was highly significant (Fg × hs = 4.00, *p* < 0.00001), indicating the presence of clones with distinct yield performance over time (Figure 1). In turn, the high repeatability estimate indicates that some clones maintained productive stability across harvest seasons (r > 0.90). Interpreted as the upper limit of heritability, this estimate represents the proportion of total variation attributable to variance among genotypes. The low estimates of the experimental coefficient of variation, together with a ratio greater than 1 between the genetic and experimental coefficients of variation (CVg/CVe), indicate high experimental accuracy and confirm the potential for gains through selection. The experimental coefficient of variation (CVe = 21.73%) is within the range commonly reported for long-term coffee field trials and indicates acceptable experimental precision.

Principal component analysis associated with ideotypes (references of known behavior) allowed the interpretation of genotype performance over time within a single analytical approach. The first component (PC1 = 73.90%) highlighted the contrast between maximum and minimum adaptability (ideotypes I and IV) and primarily represented differences in mean yield among genotypes, whereas the second (PC2 = 18.90%) revealed patterns of specific adaptation associated with temporal yield oscillation and biennial bearing. This distribution indicates that yield potential was more decisive than inter-harvest oscillation in the selection of superior genotypes. Clones such as BAG19, BRS1216, GJ8, GJ25, AS2, and BAG24 were positioned close to ideotype I (high yield and yield stability), whereas other genotypes were associated with ideotypes II and III (marked oscillation) or IV (low productivity) (Figure 1).

**Figure 2:**
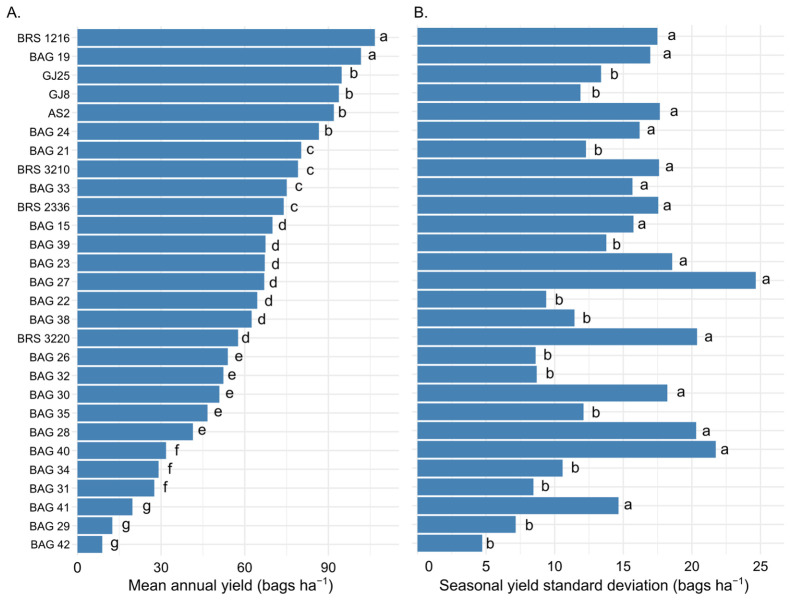
Mean annual yield (**A**) and seasonal yield standard deviation (**B**) of *Coffea canephora* genotypes, expressed in 60 kg bags ha^−1^. The standard deviation was calculated across five harvest seasons and used as a measure of interannual yield variability. Lower values indicate greater production stability. Genotypes were ordered in descending mean yield (**A**) and grouped using the Scott–Knott test at the 5% significance level (*p* ≤ 0.05). Different letters indicate statistically significant differences among groups.

In the comparison of annual productivity, clones BRS1216 and BAG19 (a) comprised the superior group, whereas GJ25, GJ8, AS2, and BAG24 formed the second stratum (b), while BAG29 and BAG42 showed the lowest means (g) (Figure 2). Regarding yield oscillation across harvest seasons, most genotypes were classified in the group with greater variation (a), whereas a smaller set exhibited lower oscillation (b), including GJ25 and GJ8, which therefore combined high yields with greater stability. Clones BRS1216 and BAG19 were highly productive but showed greater oscillation (Figure 2).

**Figure 3:**
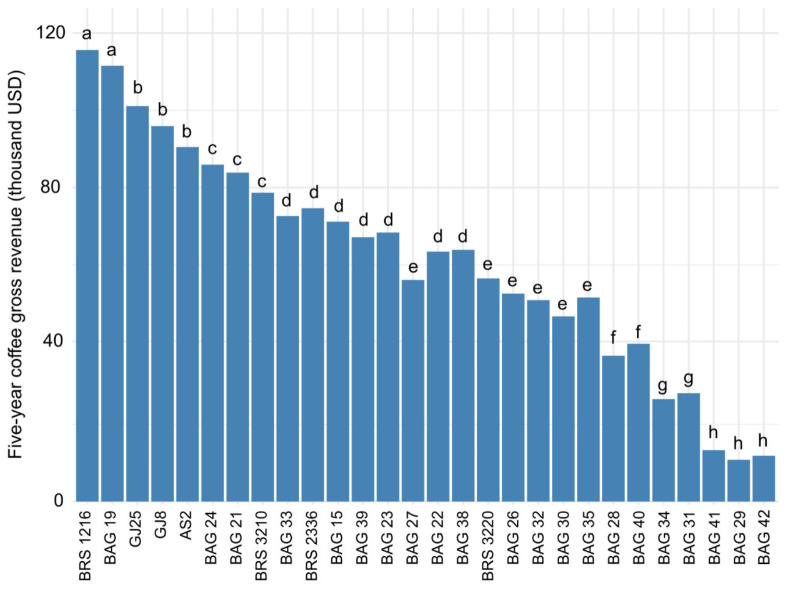
Five-year coffee gross revenue of *Coffea canephora* genotypes, expressed in thousand USD. Clones were ordered in descending revenue and grouped using the Scott–Knott test at the 5% significance level (*p* ≤ 0.05). Distinct letters indicate statistically significant differences among groups.

**Figure 4:**
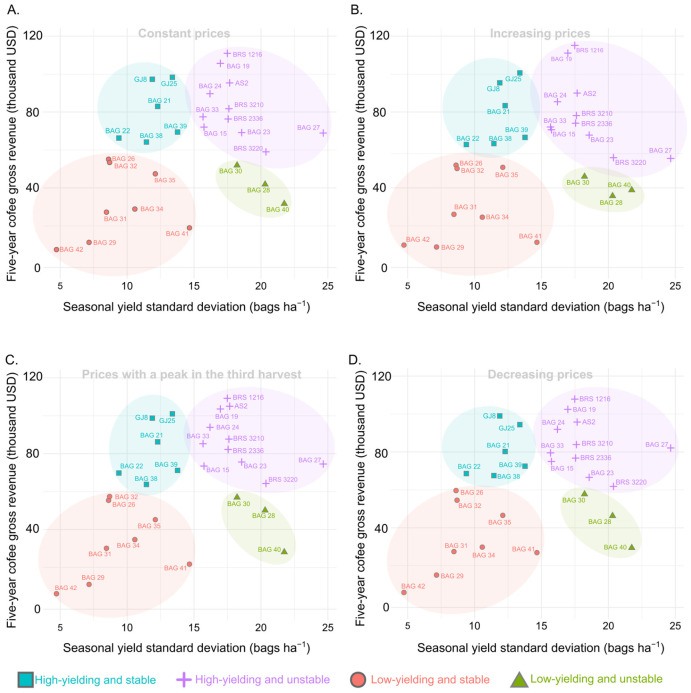
Principal component analysis (PCA) of *Coffea canephora* genotypes based on gross revenue (thousand USD) and seasonal yield variation (bags ha^−1^) under four coffee price scenarios: (**A**) constant prices, (**B**) ascending prices, (**C**) peak in the third harvest, and (**D**) descending prices. Genotypes were classified according to the yield level and yield stability, as follows: high-yielding and stable (cyan), high-yielding and unstable (purple), low-yielding and stable (red), and low-yielding and unstable (green). Ellipses represent the dispersion of each classification group.

The economic evaluation under different price scenarios revealed relevant contrasts among clone groups (Table 1, Figure 3). Under the constant price scenario, genotypes classified as high-yielding and stable showed the highest accumulated gross revenue, followed by high-yielding and unstable genotypes. Under increasing prices or a price peak in the third harvest, a relative increase in revenue was observed for unstable genotypes, whereas under the decreasing price scenario, the advantage remained with high-yielding and stable materials (Figure 4). Despite these variations, clones with the highest mean yield remained superior across all scenarios, indicating that yield potential exerted a greater influence on gross revenue than yield oscillation across harvest seasons. The maintenance of similar clone rankings under uniformly low or high price scenarios indicates that cumulative yield remained the main factor associated with gross revenue. In contrast, under scenarios with temporal variation in coffee prices, the effect of production oscillation became proportionally more important, particularly among unstable genotypes.

The Scott–Knott groupings obtained for gross revenue closely reflected those observed for mean yield. The six genotypes classified among the highest-yielding groups (BRS1216, BAG19, GJ25, GJ8, AS2, and BAG24) also ranked among the highest-revenue genotypes, indicating strong convergence between biological and economic performance. This agreement demonstrates that yield potential was the primary determinant of economic return across all evaluated price scenarios. In general, genotypes with greater productive stability tended to show lower economic variation among scenarios. In contrast, clones such as BAG42 and BAG29 combined low yield with greater seasonal yield variation, resulting in consistently lower revenues under all evaluated scenarios.

The simulation of cumulative gross revenue over five harvest seasons highlighted contrasts among price scenarios and clone groups (Table 2). Among high-yielding genotypes, yield-stable clones showed lower sensitivity to price fluctuations, with losses of up to −1.3% under the increasing price scenario and moderate gains (+2.5%) under the peak scenario. High-yielding but unstable clones, although exhibiting greater yield variability, achieved the highest increase in revenue under the peak scenario (+5.3%). For low-yielding groups, despite lower cumulative revenue, instability provided a relative advantage under variable price scenarios, resulting in gains of up to +7.4% compared to the constant price scenario. These results indicate that the combination of high yield and yield stability maximizes gross revenue under constant or declining price conditions, whereas unstable clones may be favored under scenarios of sharp short-term increases in coffee prices.

## 3. Discussion

Productive stability over time is a central issue in coffee cultivation, since selection based exclusively on cumulative yield may be misleading, as it may favor highly productive clones that become exhausted over time. This behavior reflects an intrinsic physiological characteristic of the species, in which high fruiting in one year reduces the emission of vegetative branches in the subsequent cycle. Coffee reproductive cycles are strongly regulated by phenological dynamics and environmental conditions, which directly affect vegetative growth, flowering, fruiting, and resource allocation patterns across successive harvest seasons [9]. In this context, fluctuations in productivity may be related to competition for the assimilation between reproductive demand and vegetative recovery, contributing to the biennial bearing behavior commonly observed in coffee crops.

In field-grown plants, DaMatta et al. [10] observed that the source–sink relationship between leaves and fruits depends on a minimum leaf area of 13.4 cm^2^ per fruit to sustain development under competition for photoassimilates between fruits and vegetative tissues. In *C. arabica*, Chaves et al. [11] also observed that reductions in the leaf-to-fruit ratio compromise growth and lead to branch decline. Therefore, when plants allocate a large proportion of their resources to high fruit production in one year, reduced investment in vegetative structures capable of sustaining similar production in the following year is expected.

Some studies suggest that these yield oscillations across harvest seasons may be intensified under climate change scenarios [12]. However, other studies indicate that the relationship between climatic factors and yield fluctuations is not as direct as previously assumed, as yield losses may be mitigated by several factors, such as the use of improved varieties [13,14].

The evaluation of yield over five harvest seasons revealed consistent differences among clones, allowing for the identification of superior genotypes. The six most productive clones (BRS1216, BAG19, GJ25, GJ8, AS2, and BAG24) showed mean yields of 93.2, 96.6, 105.9, 73.7, and 110.1 bags ha^−1^ in the 2021, 2022, 2023, 2024, and 2025 harvest seasons, respectively, with an overall mean of 95.9 bags ha^−1^ across the evaluated period. The high repeatability estimate (r > 0.90) indicates that most of the observed variation was associated with consistent differences among genotypes. This result supports the effectiveness of selection for productive stability in *C. canephora*. In perennial crops, where performance must be assessed over multiple harvest seasons, high repeatability values indicate that productive stability is strongly influenced by genetic factors, making the selection of stable and high-yielding clones particularly effective for breeding and cultivar recommendation programs.

The significance of the genotype × harvest season demonstrates that some clones did not respond uniformly over time, revealing contrasting temporal adaptation patterns. This variability results from both the environmental conditions of each year and the intrinsic differences among genotypes. From a breeding perspective, clones with greater stability tend to contrast with those with higher responsiveness [15,16]. While stable genotypes tend to ensure greater predictability and lower risk of reductions in average yield, responsive materials may be managed differently and better exploited in favorable harvest seasons. This difference in productive behavior also affected the economic response of the clones under contrasting price scenarios. While stable genotypes showed smaller variations in gross revenue, unstable materials were more responsive to temporary increases in coffee prices when periods of higher yield coincided with favorable market conditions.

Principal component analysis associated with ideotypes made it possible to interpret yield performance over time within a single analytical framework. The first component (PC1), responsible for the largest proportion of variation, highlighted the contrast between high-yielding genotypes and those with low adaptability, indicating that yield potential was a more decisive factor in genotype differentiation than yield oscillation across harvest seasons. The second component (PC2), which explained a smaller portion of the variation, captured patterns of oscillation between harvest seasons, highlighting the secondary, although not negligible, contribution of temporal stability. Clones such as BAG19, BRS1216, GJ8, and GJ25, positioned close to ideotype I, combined high yield and yield stability, characteristics that make them particularly promising for direct use or as parents in breeding programs.

Fluctuations across harvest seasons are also reported in plantations established from seed-derived seedlings when cultivated in agroforestry systems [17], reinforcing the indication that biennial bearing is an intrinsic characteristic of the species. Although bienniality is generally less pronounced in *C. canephora* than in *C. arabica*, understanding yield patterns across harvest seasons is essential both for selecting suitable genotypes and for interpreting their productive behavior over time.

The simulation of cumulative gross revenue under different price scenarios demonstrated that the combination of yield and yield stability provides greater economic security in contexts of constant or declining prices. High-yielding and stable genotypes exhibited smaller variations in revenue, ensuring greater predictability for growers. In contrast, high-yielding but unstable genotypes, although subject to greater oscillations, achieved the highest gains under scenarios of sharp price increases, revealing a profile associated with higher economic risk but also greater return potential. Among the low-yielding genotypes, instability provided a relative advantage only under variable price scenarios, without altering their lower absolute revenue levels.

The gross revenue values observed for the superior clones were associated with yield levels comparable to those reported for highly productive *C. canephora* cultivars in Brazil. Previous studies evaluating elite Conilon and Robusta materials under different production conditions have reported yield levels within a similar range [18,19,20], reinforcing the productive potential of the superior clones identified in the present study. Because gross revenue is directly influenced by yield, these results further support the economic relevance of selecting genotypes that combine high productivity and stability across harvest seasons.

The economic analysis conducted in this study was based on gross revenue and did not consider production costs, which may vary among regions and production systems. Consequently, the results should not be interpreted as estimates of net profit. Because all genotypes were evaluated under the same management conditions, including fertilization, spacing, and irrigation, the differences observed in gross revenue primarily reflected differences in the productive performance of the evaluated materials. Further studies addressing production costs could contribute to a broader understanding of the economic performance of these materials.

Taken together, the results indicate that the selection of *C. canephora* clones should prioritize materials that combine high yield potential and yield stability across harvest seasons, ensuring more consistent agronomic and economic performance over time under conditions of climatic and market variability. Although some high-yielding but unstable genotypes achieved greater gross revenue under specific simulated scenarios, these advantages occurred only when particular patterns of yield distribution coincided with favorable price conditions. Such outcomes depend on a combination of factors that cannot be predicted or controlled by growers and, therefore, should not be interpreted as a recommendation to favor instability. Market fluctuations are inherently unpredictable; therefore, yield stability remains the most reliable strategy for reducing economic risk and maintaining consistent returns over time.

Clones with these characteristics not only provide greater income predictability for farmers but also constitute valuable sources of genetic variability for breeding programs. The integration of genetic, phenotypic, and economic analyses reinforces the importance of selection strategies that simultaneously consider yield, stability, and financial return, thereby contributing to the sustainability of robusta coffee production in Brazil.

Few studies have examined the relationship between coffee production volume and price dynamics, with most focusing on identifying the factors responsible for fluctuations in coffee market prices [21,22]. These studies often converge on the need for public policies aimed at stabilizing prices to mitigate market volatility. The present study, in contrast, introduces a novel perspective by demonstrating that the selection of clones that are more adapted and stable across harvest seasons may represent an alternative strategy to cope with market oscillations. Therefore, investment in technologies aimed at developing new cultivars represents a strategy with potentially significant economic impact on the coffee sector, since the availability of cultivars with greater adaptation and productive stability may help protect coffee growers’ gross revenue in both the short and long term.

Coffee growers make production decisions under conditions in which market prices vary substantially between years. In this context, evaluating productive stability together with gross revenue under different price scenarios may contribute to the identification of clones with greater consistency of economic return over time. In the present study, the cultivation of clones such as BAG19, BRS1216, GJ8, GJ25, AS2, and BAG24 proved advantageous for ensuring high yield and gross revenue, regardless of the price scenario, considering the market fluctuations observed between 2021 and 2025 (converted to USD as described in the methodology). In addition, these clones may be jointly cultivated in commercial plantations because they belong to different compatibility groups, which may contribute to pollination efficiency and fruit set in *C. canephora* [19,20].

## 4. Materials and Methods

### 4.1. Field Experiment

The experiment was conducted at the Embrapa Experimental Field in Ouro Preto do Oeste, Rondônia, Brazil (10°43′55″ S, 62°15′19″ W; altitude 300 m). The predominant climate in the region is tropical monsoon (Am), according to the Köppen classification [23], with mean annual temperature of 25 °C and annual precipitation of approximately 2000 mm year^−1^.

The experiment was established on 28 February 2019, using a spacing of three meters between rows and one meter between plants (3 m × 1 m), with plants trained with two stems. The same spacing was adopted for all genotypes to ensure standardized field conditions and direct comparison among materials. The trial included hybrid clones derived from the Active Germplasm Bank (BAG) of *C. canephora* maintained by Embrapa Rondônia, as well as registered cultivars and commercially available public-domain clones.

The experiment consisted of 28 treatments (Table 3), including 21 BAG accessions, four registered cultivars, and three clones with no defined genetic origin that are commercially available in the public domain. The public-domain clones GJ8 and GJ25 originated from selections made by the coffee grower Geraldo Jacomin in Nova Brasilândia, Rondônia, Brazil, whereas AS2 originated from a selection made by the coffee grower Ademar Schmidt in Alta Floresta d’Oeste, Rondônia, Brazil. These materials are commercially used in the region and were included as local reference clones. The experimental design was a randomized complete block design with four replications. Each experimental plot consisted of five plants, with border rows to minimize edge effects, totaling 600 plants in the experiment and occupying an area of 1800 m^2^ (0.18 ha).

**Figure 5:**
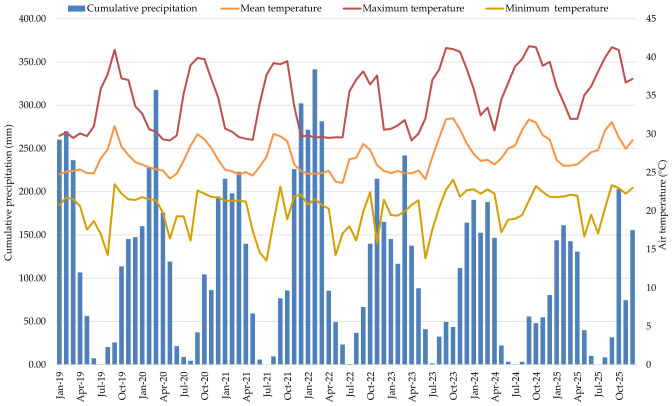
Monthly cumulative precipitation and air temperature (mean, minimum, and maximum) during the experimental period from 2019 to 2025 in Ouro Preto do Oeste, Rondônia, Brazil, based on NASA POWER data [23].

Monthly cumulative precipitation and air temperature (minimum, mean, and maximum) from planting to the last harvest are presented in Figure 5. Meteorological data were obtained from the NASA POWER database [24] for the municipality where the experiment was conducted.

Supplemental irrigation was applied using a drip irrigation system with two emitters per plant, each with a flow rate of 1.6 L h^−1^. During the dry period (June–September), irrigation was applied according to crop water demand. From planting until the first flowering (July 2020), irrigation was applied for 2 h day^−1^, corresponding to 6.4 L plant^−1^ day^−1^ (2.13 mm day^−1^). After this period, the irrigation time was increased to 3 h day^−1^, corresponding to 9.6 L plant^−1^ day^−1^ (3.20 mm day^−1^).

Crop management practices followed technical recommendations for coffee cultivation under Amazonian conditions [25], including weed control through herbicide application and mechanical mowing, phytosanitary management based on periodic monitoring of pests and diseases, and plant nutritional management. Mineral fertilizer rates were uniformly applied to all genotypes and were defined based on soil chemical analysis and the expected mean yield of the experimental population. This standardized fertilization strategy was adopted to allow direct comparison among genotypes under the same management conditions and to avoid confounding genotype performance with genotype-specific fertilization.

Soil chemical properties of the experimental area were determined at depths of 0–20 cm (2019 A), 20–40 cm (2019 B), and 40–60 cm (2019 C) in January 2019, prior to the establishment of the experiment, and subsequently monitored during the experimental period (2019–2024), with the analysis corresponding to 2024 presented (Table 4).

The soil of the area, previously under fallow, was not prepared over the entire area. Soil preparation was restricted to planting holes. The holes were opened using a tractor-mounted soil auger. The planting holes were prepared with dimensions of 1.0 m in depth and 0.7 m in diameter, corresponding to a soil volume of 0.380 m^3^ per hole.

During hole preparation, the following soil amendment and fertilization practices were carried out: 100 g of agricultural gypsum was applied at the bottom of each hole; 380 g of dolomitic lime was distributed along the walls and mixed with the soil; 300 g of natural phosphate, corresponding to approximately 60 g of P_2_O_5_, was incorporated into the bottom, walls, and soil during mixing; 5 L of coffee straw were added and thoroughly mixed with the soil; 300 g of triple superphosphate, corresponding to approximately 123 g of P_2_O_5_, were applied; and, finally, 50 g of micronutrients (MIB), containing 3.0% S, 2.5% B, 1.0% Cu, 0.1% Mo, 10.0% Mn, and 7.0% Zn, were placed in the lower half of the hole to avoid direct contact with the seedlings at planting.

After the coffee plants were planted, soil acidity correction was performed through the application of dolomitic lime (PRNT 90%) at rates of 200 g plant^−1^ in 2019, 2020, and 2021, and 600 g plant^−1^ in 2022. Lime was applied within a 1.0 m-wide band along the planting row and extending 1.0 m toward the inter-row, covering 0.5 m on each side of the plant. The remaining macro- and micronutrients were supplied through manual surface applications at 30-day intervals, except during the months of May and June due to fruit maturation and harvest. Fertilizer rates were calculated to meet crop demand according to the recommendations of Marcolan et al. [25].

Annual fertilization rates varied according to crop demand and soil analysis. Nitrogen (N) was applied at 229, 401, 401, 330, 275, and 282 kg ha^−1^ in 2019, 2020, 2021, 2022, 2023, and 2024, respectively. Phosphorus (P_2_O_5_) was applied at 51, 117, 117, 111, 50, and 68 kg ha^−1^, while potassium (K_2_O) was applied at 67, 293, 293, 167, 260, and 200 kg ha^−1^ during the same years.

The agronomic traits evaluated were outturn index (%) and yield (bags ha^−1^) during the 2021–2025 harvest seasons. The outturn index was evaluated because coffee commercialization is based on processed coffee rather than fresh fruit mass. Therefore, this trait provides complementary information to field yield and allows a more accurate estimation of the amount of marketable coffee produced by each genotype.

Harvesting was performed when approximately 80% of the fruits were ripe. After harvesting, cherry coffee was washed, separated, and naturally dried in elevated drying structures for 10 to 15 days until reaching approximately 12% moisture, measured using a Gehaka grain moisture meter (G600).

Subsequently, 250 g samples of dried cherry coffee were processed using a manual pulping machine, followed by separation with a set of sieves. Outturn index was calculated based on the weight reduction observed during drying and processing, adjusted to 12% moisture [26]. It was estimated using the following expression:

$$Outturn\;index\left(\%\right)=\frac{Mdry\;cherry}{Mfrom\;field}\times \frac{Mbeans}{Mbeans+Mhusk}\times 100\times F_{moist\;12\%}$$

where Outturn index (%): ratio corresponding to the mass of processed coffee relative to the fresh coffee mass; *Mdry cherry*: mass of dry cherry coffee; *Mfrom field*: mass of the coffee from the field; *Mbeans*: mass of the coffee beans; *Mbeans* + *Mhusk*: total mass of the dried fruits before processing, including both coffee beans and husk; and *F_moist_*_12%_: correction factor used to standardize coffee mass to 12% moisture, calculated from the ratio between the observed moisture content and the reference moisture content of 12%.

Yield was evaluated over five consecutive harvest seasons (2021–2025), estimated as bags of processed coffee per hectare (bags ha^−1^). Ripe fruits were harvested per plant and subsequently weighed using a precision scale. The values were converted from harvested fresh coffee to 60 kg bags of processed coffee per hectare using the following equation:

$$Yield=\frac{\left(\frac{CR}{NP}\right)}{60}\times nplant\times Outturn\;index$$

where Yield: coffee yield (bags ha^−1^); *CR*: coffee production per plot (kg); *NP*: number of plants per plot; *nplant*: plant density (plants ha^−1^); *Outturn index*: ratio (%) corresponding to the mass of processed coffee equivalent to one 60 kg bag.

The mean yield across the five harvest seasons was used as the main response variable, together with cumulative yield. Additionally, yield oscillation (Oscillation) was estimated as the standard deviation of yield among harvest seasons and used as a measure of interannual yield variability. Lower values indicate greater yield stability over time:

$${Oscillation}_{i}=\sqrt{\frac{\sum_{j=1}^{5}{\left(Y_{ij}-\;{\bar{Y}}_{i}\right)}^{2}}{n-1}}$$

where *Oscillation*_i_ is the seasonal yield standard deviation of the *i*-th plot across the five harvest seasons (*j*); *Y_ij_* is the yield observed in the *j*-th harvest; *Y_i_* is the mean yield of the *i*-th plot across the five harvest seasons; and n is the number of harvest seasons evaluated.

Gross revenue from the commercialization of the produced coffee was also used as a response variable. Gross revenue was estimated considering annual average prices, calculated from the product of the number of bags produced in each year and the average market price recorded for the respective year, according to data from the Vitória Coffee Trade Center and the Brazilian Coffee Industry Association [27,28]. The average prices recorded were R$ 516.59 per 60 kg bag in 2021, R$ 649.83 in 2022 and 2023, R$ 1500.00 in 2024, and R$ 1973.25 in 2025 (up to August).

All economic values were initially calculated in Brazilian currency (R$), as coffee commercialization in the study region is conducted in Brazilian reais. Subsequently, the values were converted to U.S. dollars (USD) using a fixed average exchange rate of R$ 5.10 per USD for the 2021–2025 period. This conversion was adopted to facilitate international interpretation of the results and to avoid introducing additional variation associated with annual exchange-rate fluctuations. After conversion, the average prices were USD 101.29 per bag in 2021, USD 127.42 per bag in 2022 and 2023, USD 294.12 per bag in 2024, and USD 386.91 per bag in 2025.

The price scenarios were used as retrospective simulations to evaluate how differences in yield distribution across harvest seasons would affect gross revenue among genotypes under different price conditions.

Gross revenue was then evaluated under four different scenarios regarding price variability across the five years:

(a) Constant prices: Assuming that the average price (USD 207.43) was paid per 60 kg bag of coffee in all five years, without variation.
(b) Increasing prices, with lower values paid per bag in the first year and higher values in the fifth year, corresponding to the observed market scenario: USD 101.29 per bag in 2021; USD 127.42 per bag in 2022 and 2023; USD 294.12 per bag in 2024; and USD 386.91 per bag in 2025.
(c) Prices with a peak in the third harvest: USD 101.29 per bag in 2021; USD 127.42 per bag in 2022; USD 386.91 per bag in 2023; USD 294.12 per bag in 2024; and USD 127.42 per bag in 2025.
(d) Decreasing prices, with higher values paid per bag in the first year and lower values in the final year: USD 386.91 per bag in 2021; USD 294.12 per bag in 2022; USD 127.42 per bag in 2023 and 2024; and USD 101.29 per bag in 2025.

### 4.2. Statistical Analyses

Genotype effects and the genotype × harvest season interaction were evaluated by analysis of variance to quantify the effect of the genotype × harvest season interaction according to the model described by Cruz, Carneiro, and Bhering [29]:

$$Y_{ijk}=\mu +G_{i}+A_{j}+B_{k(j)}+GA_{ij}+e_{ijk}$$

where *Y_ijk_* refers to the observation of the *i*-th genotype in the *j*-th harvest season; μ is the experimental mean; *G_i_* is the effect of the *i*-th genotype; *A_j_* is the effect of the *j*-th harvest season; *B_k_*_(*j*)_ is the effect of block *k* within environment *j* (blocks nested within environment); *GA_ij_* is the interaction effect between genotypes and harvest seasons; and *e_ijk_* is the experimental error.

Estimates of the F test from the analysis of variance, the experimental coefficient of variation (CVe), the genetic coefficient of variation (CVg), and repeatability were interpreted. Means were grouped using the Scott–Knott clustering test (*p* ≤ 0.05).

The centroid method was used to estimate stability and adaptability, considering vector data of maximum and minimum genotype performances in each harvest season. These vectors provided references of the “ideal” minimum (min), medium (med), and maximum (max) performances of the genotypes in favorable (f) and unfavorable (uf) harvest season [30].

The clones were classified based on the Euclidean distance between each genotype and the behavioral reference points (centroids), according to the following model:

$$D_{ik}=\sqrt{\sum_{j=1}^{n}(x_{ij}-c_{jk}{)}^{2}}$$

where *D_ik_* is the Euclidean distance from the *i*-th genotype to the *k*-th centroid (*k* = 1, 2, …, *n*); *x_ij_* is the performance of the *i*-th genotype in the *j*-th harvest season; and $c_{jk}$ is the performance of the *k*-th centroid in the *j*-th harvest season. Based on these distances (D_ik_), the genotypes were classified as follows: I = high overall adaptability (max(f) and max(uf)), II = adaptability specific to favorable environments (max(f) and min(uf)), III = adaptability specific to unfavorable environments (min(f) and max(uf)), IV = low adaptability (min(f) and min(uf)), V = medium overall adaptability (med(f) and med(uf)), VI = adaptability specific to favorable environments (max(f) and med(uf)), and VII = adaptability specific to unfavorable environments (med(f) and max(uf)).

From the expected mean square estimates, repeatability was estimated as follows [28]:

$$r=\frac{C\hat{O}V(Y_{ij}Y_{{ij}^{\prime }})}{\sqrt{\hat{V}\left(Y_{ij}\right)\hat{V}(Y_{{ij}^{\prime }})}}=\frac{\sigma_{p}^{2}}{\sigma_{p}^{2}+\sigma_{et}^{2}}$$

where *r* is the repeatability coefficient; $\sigma_{p}^{2}$ is the genotypic variance combined with the variance of permanent environmental effects; $\sigma_{et}^{2}$ is the temporary environmental variance associated with experimental error.

Estimates of the experimental coefficient of variation and the genetic coefficient of variation were used to assess experimental precision. Clone performance was evaluated through scatter plots and repeatability estimates across harvest seasons. Genotypes were then classified according to their performance using the Scott–Knott test at a 5% significance level.

Based on this grouping, clones were classified into two stability levels according to the results for yield oscillation. Scatter plots were generated relating gross revenue, considering annual average prices, to yield oscillation, with clones identified according to productivity and stability levels.

All statistical analyses were performed using R software version 4.5.2 via the RStudio interface [31].

## 5. Conclusions

The long-term evaluation (five harvest seasons) enabled the identification of *C. canephora* genotypes with high yield potential and yield stability. The significance of the genotype × harvest season interaction revealed different adaptation patterns, distinguishing stable clones from those exhibiting greater bienniality.

The combined analysis of principal components and ideotypes demonstrated that yield potential was a more decisive factor in genotype differentiation than yield oscillation across harvest seasons.

Revenue simulations under different price scenarios indicated that high-yielding and stable genotypes provided greater economic security under constant or declining price conditions. Among the evaluated materials, BAG19, BRS1216, GJ8, GJ25, AS2, and BAG24 consistently combined high yield, productive stability, and superior gross revenue across the simulated scenarios, demonstrating their potential for cultivar recommendation and use in *C. anephora* breeding programs.

Although unstable genotypes occasionally achieved higher gross revenue when years of high production coincided with favorable prices, such situations depend on unpredictable market fluctuations and should not be considered a reliable management strategy. Therefore, the selection of stable and productive genotypes remains the most consistent approach for reducing economic risk and maintaining revenue over time.

## Figures and Tables

**Table 1:** Analysis of variance (ANOVA) results for mean yield (bags ha^−1^), annual yield variation (bags ha^−1^), and revenue (thousand USD) of *Coffea canephora* genotypes across five harvests. Shown are mean squares (MS) for block, genotype, and residual effects, F-values and corresponding *p*-values, as well as the overall mean and the experimental coefficient of variation (CV%).

F Test	Mean Yield (Bags ha^−1^)	Yield Standard Deviation (Bags ha^−1^)	Revenue (Thousands USD)
MS block	58.16	14.45	211.59
MS genotype	2878.05	93.10	16,658.33
MS residual	47.45	20.34	291.23
F (genotype)	60.65	4.57	57.19
*p*-value (genotype)	<0.00001	<0.00001	<0.00001
Mean	61.25	14.49	61.52
CV(%)	11.25	31.12	12.19

MS = Mean Square; CV = Coefficient of Variation. Mean yield expressed in 60 kg bags ha^−1^; yield variation expressed in 60 kg bags ha^−1^; revenue expressed in thousand USD. The standard deviation was calculated across five harvest seasons and used as a measure of interannual yield variability.

**Table 2:** Variation in gross revenue (thousand USD ha^−1^) from coffee sales after five harvest seasons as a function of price dynamics, yield level, and adaptability of *Coffea canephora* clones.

Price Dynamics per Bag Across Five Harvest Seasons		Genotypes			
		High-Yielding and Stable	High-Yielding and Unstable	Low-Yielding and Stable	Low-Yielding and Unstable
Constant	Mean	80.07	82.67	32.51	42.92
	S.E.	±6.32	±3.77	±6.50	±9.23
Increasing *	Mean	79.06 (−1.3%)	79.97 (−3.3%)	30.55 (−6.0%)	41.34 (−3.7%)
	S.E.	±6.68	±4.13	±6.64	±9.92
Peak in 3rd harvest *	Mean	82.09 (+2.5%)	87.00 (+5.3%)	34.01 (+4.6%)	46.11 (+7.4%)
	S.E.	±6.76	±4.05	±6.66	±9.42
Decreasing	Mean	80.69 (+0.7%)	84.22 (+1.9%)	34.50 (+6.1%)	45.55 (+6.1%)
	S.E.	±6.11	±3.79	±6.53	±8.84

* Coffee prices per 60 kg bag were based on values recorded from 2021 to 2025, according to data from the Vitória Coffee Trade Center and the Brazilian Coffee Industry Association (ABIC): R$ 516.59 (2021), R$ 649.83 (2022 and 2023), R$ 1500.00 (2024), and R$ 1973.25 (2025, up to August). Gross revenue was calculated in Brazilian currency (R$) and converted to U.S. dollars (USD) using a fixed exchange rate of R$ 5.10 per USD. Price scenarios included constant, increasing, peaking in the third harvest season, and decreasing trends. Values in parentheses indicate percentage variation relative to the constant price scenario. S.E.—standard error.

**Table 3:** Identification, status, genealogy, and origin of the 28 *Coffea canephora* genotypes evaluated in the field experiment in Ouro Preto do Oeste, Rondônia, Brazil.

n	Genotype	Status	Genealogy	Origin
1	BRS 1216	Registered cultivar	Emcapa03 × Robusta1675	Embrapa
2	BRS 2336	Registered cultivar	Open pollination	Embrapa
3	BRS 3210	Registered cultivar	Emcapa03 × Robusta2258	Embrapa
4	BRS 3220	Registered cultivar	Emcapa03 × Robusta1675	Embrapa
5	GJ8	Public domain	Open pollination	Geraldo Jacomin ^1^
6	GJ25	Public domain	Open pollination	Geraldo Jacomin ^1^
7	AS2	Public domain	Open pollination	Ademar Schmidt ^2^
8	BAG 15	Embrapa collection	Emcapa03 × Robusta2258	Embrapa
9	BAG 19	Embrapa collection	Emcapa03 × Robusta1675	Embrapa
10	BAG 21	Embrapa collection	Robusta1675 × Cpafro194	Embrapa
11	BAG 22	Embrapa collection	Emcapa03 × Robusta2258	Embrapa
12	BAG 23	Embrapa collection	Open pollination	Embrapa
13	BAG 24	Embrapa collection	Emcapa03 × Robusta1675	Embrapa
14	BAG 26	Embrapa collection	Emcapa03 × Robusta2258	Embrapa
15	BAG 27	Embrapa collection	Emcapa03 × Robusta2258	Embrapa
16	BAG 28	Embrapa collection	Open pollination	Embrapa
17	BAG 29	Embrapa collection	Open pollination	Embrapa
18	BAG 30	Embrapa collection	Open pollination	Embrapa
19	BAG 31	Embrapa collection	Open pollination	Embrapa
20	BAG 32	Embrapa collection	Open pollination	Embrapa
21	BAG 33	Embrapa collection	Open pollination	Embrapa
22	BAG 34	Embrapa collection	Open pollination	Embrapa
23	BAG 35	Embrapa collection	Open pollination	Embrapa
24	BAG 38	Embrapa collection	Open pollination	Embrapa
25	BAG 39	Embrapa collection	Open pollination	Embrapa
26	BAG 40	Embrapa collection	Open pollination	Embrapa
27	BAG 41	Embrapa collection	Open pollination	Embrapa
28	BAG 42	Embrapa collection	Open pollination	Embrapa

^1^ Coffee grower from the municipality of Nova Brasilândia, Rondônia, Brazil. ^2^ Coffee grower from the municipality of Alta Floresta d’Oeste, Rondônia, Brazil.

**Table 4:** Chemical properties of the *Argissolo Vermelho-Amarelo* soil in the clonal garden of the experimental field of Embrapa in Ouro Preto do Oeste, Rondônia, Brazil, in 2019–2024.

Ano	pH	OM	P	K	Ca	Mg	H + Al	Al	CEC	m	V
	Water	g kg^−1^	mg dm^−3^	cmol_c_ dm^−3^						%	
2019 A	6.1	16.7	12	0.87	2.97	0.97	4.19	0.0	9.00	0.0	53
2019 B	6.3	11.8	8	0.34	2.35	0.73	3.22	0.0	6.64	0.0	52
2019 C	6.3	5.4	5	0.20	2.06	0.70	2.34	0.0	5.33	0.0	56
2020 A	5.6	13.9	28	0.82	2.39	1.19	4.37	0.0	8.76	0.0	50
2020 B	6.0	12.0	21	0.35	3.03	0.89	2.89	0.0	7.15	0.0	60
2021 A	5.1	11.1	44	0.77	1.80	1.40	4.54	0.0	8.51	0.0	47
2021 B	5.7	8.1	34	0.35	3.70	1.04	2.56	0.0	7.65	0.0	67
2022 A	5.3	10.9	40	0.48	2.13	1.30	4.15	0.0	8.06	0.0	49
2022 B	5.7	6.5	25	0.24	2.77	0.90	2.70	0.0	6.61	0.0	58
2023 A	5.4	10.5	45	0.18	2.45	1.20	3.76	0.0	7.60	0.0	50
2023 B	5.6	4.4	16	0.13	1.84	0.75	2.84	0.0	5.56	0.0	49
2024 A	6.2	19.4	15	0.14	3.15	1.57	2.67	0.0	7.42	0.0	65
2024 B	5.5	3.5	7	0.02	0.91	0.46	3.12	0.0	4.51	0.0	30

Soil pH was measured using water at a ratio of 1:2.5. OM: organic matter (wet digestion); P: phosphorus; K: potassium; Ca: calcium; Mg: magnesium; Al: aluminum. P and K contents were determined using the Mehlich 1 method. Exchangeable Ca, Mg, and Al were extracted with 1 mol dm^−3^ KCl. H + Al: potential acidity; CEC: cation exchange capacity; V: base saturation; m: Al^3+^ saturation. Notes: A = 0–20 cm; B = 20–40 cm; and C = 40–60 cm.

## Data Availability

The data supporting the findings of this study are available in the article.

## Abbreviations

The following abbreviations are used in this manuscript:

ABIC	Brazilian Coffee Industry Association
BAG	Active Germplasm Bank
BRS	Cultivars released by Embrapa
CCCV	Centro do Comércio de Café de Vitória
CV	Coefficient of Variation
CVe	Experimental Coefficient of Variation
CVg	Genotypic Coefficient of Variation
NASA	National Aeronautics and Space Administration
PCA	Principal Component Analysis
PC1	First Principal Component
PC2	Second Principal Component
PRNT	Relative Neutralizing Power Total
USD	United States Dollar

## References

[1] Ahmed S., Brinkley S., Smith E., Sela A., Theisen M., Thibodeau C., Cash S.B. (2021). Climate change and coffee quality: Systematic review on the effects of environmental and management variation on secondary metabolites and sensory attributes of *Coffea arabica* and *Coffea canephora*. Front. Plant Sci..

[2] Tournebize R., Borner L., Manel S., Meynard C.N., Vigouroux Y., Crouzillat D., Poncet V. (2022). Ecological and genomic vulnerability to climate change across native populations of Robusta coffee (*Coffea canephora*). Glob. Change Biol..

[3] Custodio A.M., Menezes Silva P.E., Santos T.R.D., Lourenço L.L., Avila R.G., Silva A.R., Silva F.G. (2022). Seasonal variation in physiological traits of Amazonian *Coffea canephora* genotypes under contrasting water availability. Agronomy.

[4] Senra J.F.B., Silva V.A.C., Esposti M.D.D., Ferreira A., Milheiros I.S., Ramos I.B., Oliveira R.G., Benevenute L.M. (2025). Selection of differentiated maturity genotypes of *Coffea canephora*. Acta Sci. Agron..

[5] Partelli F.L., da Silva F.A., Covre A.M., Oliosi G., Correa C.C.G., Viana A.P. (2022). Adaptability and stability of *Coffea canephora* to dynamic environments using the Bayesian approach. Sci. Rep..

[6] Adunola P., Ferrão M.A.G., Ferrão R.G., Fonseca A.F.A., Volpi P.S., Comério M., Verdin Filho A.C., Muñoz P.R., Ferrão L.F.V. (2023). Genomic selection for genotype performance and environmental stability in *Coffea canephora*. G3 Genes Genomes Genet..

[7] Turco P.H.N., Esperancini M.S.T., Bueno O.C., Oliveira M.M. (2017). Economic profitability in conventional and irrigated coffee production systems in three municipalities in the Marília region of São Paulo, Brazil. Cienc. Rural.

[8] Carpio C.E., Sandoval L.A., Munoz M. (2023). Cost and profitability analysis of producing specialty coffee in El Salvador and Honduras. HortTechnology.

[9] Unigarro C.A., Cayón-Salinas D.G., Leão-Burgos A.F., Flórez-Ramos C.P. (2025). Flowering and fruiting of *Coffea arabica* L.: A comprehensive perspective from phenology. Plants.

[10] DaMatta F.M., Ronchi C.P., Maestri M., Barros R.S. (2008). In field-grown coffee trees, source–sink manipulation alters photosynthetic rates, independently of carbon metabolism, via alterations in stomatal function. New Phytol..

[11] Chaves A.R.M., Martins S.C.V., Batista K.D., Celin E.F., DaMatta F.M. (2012). Varying leaf-to-fruit ratios affect branch growth and dieback, with little to no effect on photosynthesis, carbohydrate or mineral pools, in different canopy positions of field-grown coffee trees. Environ. Exp. Bot..

[12] Chengappa P.G., Devika C.M., Rudragouda C.S. (2017). Climate variability and mitigation: Perceptions and strategies adopted by traditional coffee growers in India. Clim. Dev..

[13] Bacsi Z., Fekete-Farkas M., Ma’ruf M.I. (2022). Coffee yield stability as a factor of food security. Foods.

[14] Bertrand B., Mieulet D., Breitler J.C., Leroy T., Montagnon C. (2025). Breeding of new coffee varieties as a key strategy to improve coffee sustainability in response to climate change. Advances in Botanical Research.

[15] Souza C.A.D., Teixeira A.L., Torres J.D., Silva C.A., Espindula M.C., Rocha R.B. (2019). Adaptability and stability of *Coffea arabica* lines in the Western Amazon. Coffee Sci..

[16] Moraes M.S., Rocha R.B., Teixeira A.L., Espindula M.C., Silva C.A., Lunz A.M.P. (2020). Adaptability and stability of *Coffea canephora* Pierre ex Froehner genotypes in the Western Amazon. Cienc. Rural.

[17] Bezerra S.B.O., Araújo L.F.B., Costa R.S.C., Souza V.F., Rocha R.B., Campanharo M., Espindula M.C. (2024). Growing *Coffea canephora* in agroforestry systems with Brazilian firetree, Brazil nut, and teak. Semin. Cienc. Agrar..

[18] Bergo C.L., Miqueloni D.P., Lunz A.M.P., Assis G.M.L.d. (2020). Estimation of genetic parameters and selection of *Coffea canephora* progenies evaluated in Brazilian Western Amazon. Coffee Sci..

[19] Teixeira A.L., Rocha R.B., Espindula M.C., Ramalho A.R., Vieira Júnior J.R., Alves E.A., Lunz A.M.P., Souza F.F., Costa J.N.M., Fernandes C.F. (2020). Amazonian robustas—New *Coffea canephora* coffee cultivars for the Western Brazilian Amazon. Crop Breed. Appl. Biotechnol..

[20] Silva A.N.R., Rocha R.B., Teixeira A.L., Espindula M.C., Partelli F.L., Caixeta E.T. (2024). Self-incompatibility and pollination efficiency in *Coffea canephora* using fluorescence microscopy. Agronomy.

[21] Rice R. (2003). Coffee production in a time of crisis: Social and environmental connections. SAIS Rev..

[22] Bastianin A., Lanza A., Manera M. (2018). Economic impacts of El Niño Southern Oscillation: Evidence from the Colombian coffee market. Agric. Econ..

[23] Alvares C.A., Stape J.L., Sentelhas P.C., Moraes J.L.G., Sparovek G. (2013). Köppen’s climate classification map for Brazil. Meteorol. Z..

[24] NASA POWER Project NASA Prediction of Worldwide Energy Resources. https://power.larc.nasa.gov/.

[25] Marcolan A.L., Espindula M.C. (2015). Café na Amazônia.

[26] Silva A.N.R., Rocha R.B., Moraes A.D.O., Espindula M.C., Teixeira A.L., Alves E.A. (2025). Unraveling the genetic diversity of coffee processing traits in *Coffea canephora*. Cienc. Rural.

[27] Brazilian Coffee Industry Association (ABIC) Historical Prices Paid to Coffee Producers. https://www.abic.com.br/estatisticas/preco-pago-ao-produtor/.

[28] Centro do Comércio de Café de Vitória (CCCV) Historical Coffee Prices. https://www.cccv.org.br/cotacao/cotacoes-antigas/.

[29] Cruz C.D., Carneiro P.C.S., Bhering L.L. (2021). Biometry in plant breeding. Crop Breed. Appl. Biotechnol..

[30] Rocha R.B., Muro-Abad J.I., Araújo E.F., Cruz C.D. (2005). Avaliação do método centróide para estudo de adaptabilidade ao ambiente de clones de *Eucalyptus grandis*. Cienc. Florest..

[31] R Core Team (2025). R: A Language and Environment for Statistical Computing.

